# Supplementary material for: Utility and use of accuracy cues in social learning of crowd preferences
Source: PLoS One. 2020 Oct 28;15(10):e0240997. doi: 10.1371/journal.pone.0240997 (PMC7592789; doi:10.1371/journal.pone.0240997)
Supplement: S1 Text — (DOCX) [file pone.0240997.s001.docx]

S1 Text

**Details of the hierarchical regression model**

**Hierarchical regression model for human use of accuracy cues**

Considering that the parameters for the 6 model terms ($B_{i}^{'}, W_{0 i}^{'}, \beta_{cm i}^{'}, \beta_{n i}^{'}, \beta_{a i}^{'}, \beta_{co i}^{'},$where $i$ denotes an individual and the apostrophe was used to indicate that they are the parameters of an individual) are likely to differ across individuals and such across-individual variabilities can be correlated among the parameters, we assumed that a vector of 6 individual parameters is a sample from a multivariate normal distribution with the mean vector, $\boldsymbol{M}=[ B_{i}, W_{0}, \beta_{cm},\beta_{n}, \beta_{a}, \beta_{co}]$, and the covariance matrix, $\boldsymbol{\Sigma}$. We also assumed that the random behavioral variability follows a beta binomial distribution. Accordingly, a revised SP estimate for given individual $i$ and item $j$, ${\hat{SP}^{r}}_{ij}$, was modeled as a random sample from a beta binomial distribution with an expected value, $\mu_{ij}$. The range of $\mu_{ij}$ was bounded to between 1.2 and 23.8 by a truncating function $\boldsymbol{f}$ (the use of slightly different bounding values did not affect the results qualitatively). Based on these assumptions, we built a hierarchical regression model (HRM) for human social revision of SP estimates as follows:

- ${\hat{SP}^{r}}_{ij} \sim betabinom \left( mean=\mu_{ij}-1, n=23, precision= k \right)+1$
- $\mu_{ij}= \boldsymbol{f} [ {\hat{SP}^{m}}_{ij}+W_{ij} {{(\hat{SP}}^{o}}_{ij}-{\hat{SP}^{m}}_{ij}) +B_{i}^{'} ]$ (Eq. S1A)
- $W_{ij}=W_{0 i}^{'}+{\beta'}_{cm i} {c^{m}}_{ij}+{\beta'}_{n i} {n^{o}}_{ij}+{\beta'}_{a i} {a^{o}}_{ij} +{\beta'}_{co i} {c^{o}}_{ij}$
- $\left[ B_{i}^{'},W_{0 i}^{'},\beta_{cm i}^{'} , \beta_{n i}^{'} , \beta_{a i}^{'} ,\beta_{co i}^{'} \right] \sim MVN(mean= \boldsymbol{M}, cov= \boldsymbol{\Sigma})$
- $\boldsymbol{M}=\left[ B_{i}, W_{0}, \beta_{cm},\beta_{n}, \beta_{a}, \beta_{co} \right]$.

We estimated the parameters of the HRM, and carried out statistical significance tests on them as well, by inferring the posterior probability of $\boldsymbol{M}$ given the revised SP estimates using a Bayesian method (Figueroa-Zúñiga, Arellano-Valle, & Ferrari, 2013):

$p\left( \boldsymbol{M},\boldsymbol{\Sigma,}p\boldsymbol{|}\hat{SP}^{r} \right) \propto p\left( \hat{SP}^{r} | \boldsymbol{M},\boldsymbol{\Sigma,}p \right) p\left( \boldsymbol{M} \right) p\left( \boldsymbol{\Sigma} \right) p\left( k \right)$, (Eq. S1B)

where the priors of $\boldsymbol{M}$, $\boldsymbol{\Sigma}$, and noise precision ($k$) were set to a normal distribution (mean, 0; σ, 100; no correlations among parameters), a Wishart distribution (degree of freedom, # of parameters + 1; scaling matrix, identity matrix), and a uniform distribution (range, [0.001, 100]). We approximated $p\left( \boldsymbol{M},\boldsymbol{\Sigma,}k\boldsymbol{|}\hat{SP}^{r} \right)$ by generating the posterior samples, $\boldsymbol{[}\boldsymbol{M}_{\boldsymbol{s}},\boldsymbol{\Sigma}_{\boldsymbol{s}}\boldsymbol{,}k_{s}] \sim p\left( \boldsymbol{M},\boldsymbol{\Sigma,}k\boldsymbol{|}\hat{SP}^{r} \right)$, using a Markov Chain Monte Carlos method (Gibbs sampling by *rjags* package in R; 2 chains were used, and 30,000 samples were generated after a 50,000 burn-in period for each chain), and then calculated the expected values and their 95% confidence intervals for statistical tests (results are shown in **Fig 3a** in the main text).

**Behavioral variability (error distribution) analysis**

One of the important requirements for any successful regression models of human behavior is to use an error term that reflects closely the actual behavioral variability in a given behavioral task. In our case, this means that $\epsilon$, which represents the differences between the predicted ($\mu_{ij}$) and observed (${\hat{SP}^{r}}_{ij}$) estimates of revised SP, should be modeled by a distribution that is close to the actual error distribution in our SP revision task. Accordingly, we modeled $\epsilon$ with a beta binomial distribution by considering the following factors. First, SP estimates take discrete values, the range of which is bounded by [1, 24]. Second, the dispersion of error distribution becomes narrow near the boundary. Lastly, the shape of error distribution becomes asymmetric near the boundary (i.e., skewed).

The second and third factors were empirically grounded in the following analyses on the observed variability in a subset of trials in which others’ SP estimates were equal or very close to my initial SP estimate ( ${{{\hat{SP}^{m}}_{ij}-1\leq\hat{SP}}^{o}}_{ij}\leq{\hat{SP}^{m}}_{ij}+1$). In such trials, we could sample the random errors that cannot be ascribed to the acceptance degree $W$ as the predicted estimate of revised SP are close to *my* initial SP estimated plus the baseline bias ($\mu_{ij} \approx{\hat{SP}^{m}}_{ij}+ B$). We confirmed that the probability mass function (PMF) of revised SP estimates (${\hat{SP}^{r}}_{ij} = \mu_{ij}+\epsilon\approx{\hat{SP}^{m}}_{ij}+ B+\epsilon$) on those trials systematically changed depending on the value of ${\hat{SP}^{m}}_{ij}$ in the way a beta binomial distribution does: the PMF of ${\hat{SP}^{r}}_{ij}$ became narrow and skewed as the value of ${\hat{SP}^{m}}_{ij}$ became extreme (**Fig S1A**).

**Fig S1A Empirical distributions of SP revision errors conditional on private SP estimates.** The PMF of revised SP estimates in a selected set of trials in which others’ estimate was equal or very close to my SP estimate (${{{\hat{SP}^{m}}_{ij}-1\leq\hat{SP}}^{o}}_{ij}\leq{\hat{SP}^{m}}_{ij}+1$) is shown for each value of *my* initial SP estimates (indicated by red dashed vertical line).

**Invariance of the degree of acceptance to the difference between *my* and others’ SP estimates**

Another important assumption in our regression models is that the degree of accepting others’ SP estimates, and its modulation as well, is invariant to the absolute amount of difference between *my* and others’ SP estimates. In other words, the amount of shifting *my* initial SP estimate ($\hat{SP}^{r}-\hat{SP}^{m}$) is assumed to increase linearly as the difference between *my* and others’ SP estimates ($\hat{SP}^{o}-\hat{SP}^{m}$) increases. To empirically validate this assumption, we firstly linearly regressed the shifts ($\hat{SP}^{r}-\hat{SP}^{m}$) onto the differences ($\hat{SP}^{o}-\hat{SP}^{m}$) (as indicated by the blue lines in **Fig S1B**), then non-linearly regressed the shifts onto the differences with a smooth spline function (as indicated by the red curves in **Figure S1B**), and then checked the correspondence between the linear regression line and the smoothing spline curve. The spline curve matched the linear regression in most of the range of the differences except for those with extreme values (difference > 17; as indicated by the deviation of the red curve from the blue line around at both ends in the left panel of Figure S2). However, those seemingly ‘non-linear’ regimes were grounded on a small fraction of trials (comprising only 1.21% of the total number of trials). When those trials with extreme differences were discarded, there was a good match between the linear regression line and the spline curve, which supports the model assumption about the invariance of the degree of acceptance to the difference (distance) between *my* and others’ SP estimates.

**Fig S1B Relationship between difference and shift in data.** The relationship was assessed by two methods, linear regression and smooth spline fitting. Each transparent dot corresponds to a single trial (alpha = 0.1). The blue lines represent linear regressions, and the red curves represent smooth spline fits. The left panel shows the regression and spline fit based on the whole data. The right panel shows the regression and split fit based the trimmed data, in which the data of extreme differences (absolute difference over 17) were removed.
