# Supplementary material for: Utility and use of accuracy cues in social learning of crowd preferences
Source: PLoS One. 2020 Oct 28;15(10):e0240997. doi: 10.1371/journal.pone.0240997 (PMC7592789; doi:10.1371/journal.pone.0240997)
Supplement: S2 Text — (DOCX) [file pone.0240997.s002.docx]

S2 Text

**Alternative procedures for replicating the main findings**

**The noise-free human-learner analysis**

As stated in the Results subsection titled “**Replication of main findings with alternative procedures,”** we developed an alternative way of assessing human use of the accuracy cues for social revision of SP estimates such that it resembles the ideal learner analysis by assuming that revision occurs without any behavioral variability or individual differences as follows:

$hypothetical {\hat{SP}^{r}}_{ij}={\hat{SP}^{m}}_{ij}+W_{ij} {{(\hat{SP}}^{o}}_{ij}-{\hat{SP}^{m}}_{ij}) +B;$Eq. S2A

- $W_{ij}=W_{0}+\beta_{cm} {c^{m}}_{ij}+\beta_{n} {n^{o}}_{ij}+\beta_{a} {a^{o}}_{ij} +\beta_{co} {c^{o}}_{ij}$

where $hypothetical {\hat{SP}^{r}}_{ij}$ refers to the revised SP estimate by the hypothetical noise-free learner. The noise-free human-learner analysis is to find a set of model parameters that minimizes the deviations of the hypothetical noise-free learner’s revised estimates ($hypothetical {\hat{SP}^{r}}_{ij}$) from the humans’ revised estimates (${\hat{SP}^{r}}_{ij}$), instead of the actual SP estimates (${SP}_{j}$) as was done in the ideal-learner analysis:

- $[ {W_{0}}^{*}, {\beta_{cm}}^{*},{\beta_{n}}^{*},{\beta_{a}}^{*}, {\beta_{co}}^{*}, B^{*}] = Argmin \sum_{i,j} \left| {\hat{SP}^{r}}_{ij}-hypothetical {\hat{SP}^{r}}_{ij} \right|$. Eq. S2B

Note that **Eq. S2B** is exactly the same as the minimization rule applied in the ideal-learner analysis (**Eq. 2**) except that ${SP}_{j}$ is replaced with ${\hat{SP}^{r}}_{ij}$ and that $hypothetical {\hat{SP}^{r}}_{ij}$ and $ideal {\hat{SP}^{r}}_{ij}$ are defined by the same noise-free regression model (**Eq. S2A**). Due to this resemblance, the model parameters estimated by the noise-free human-learner analysis are directly comparable to those estimated by the ideal-learner analysis. However, this direct comparability comes at the cost of unrealistically assuming that revision of SP estimates involves neither behavioral variability nor individual differences. This was the main reason for why we preferred the HRM analysis to the noise-free human-learner analysis. But we stress that the two analyses complement each other in comparing the ideal and human uses of the accuracy cue in a robust manner.

The second row of Table S1 shows the parameters estimated by the noise-free human-learner analysis, where the 95% confidence intervals of the parameters were estimated by a non-parametric bootstrapping method (5,000 re-samplings with replacement). These parameters (orange square in **Fig S2A**) were qualitatively comparable to those estimated by the HRM analysis (orange circle in **Fig S2B**): they both matched to or deviated in the same directions from the parameters estimated by the ideal-learner. In particular, similar to what we found in the HRM analysis, the parameter for others’ confidence was not significantly different from zero as indicated by the confidence interval in Table S1 (${\beta_{co}}^{*}$= 0.001 [-0.040, 0.042])

**Fig S2A. Comparisons of the model parameters estimated by the ideal-learner, the HRM, and the noise-free human-learner analyses.** For comparison, the coefficients (parameters of model) estimated by the noise-free human-learner analysis (orange square) were juxtaposed with the parameters estimated by the ideal-learner analysis (green circle) and by the HRM analysis (orange circle with error bar), which were shown in Figure 3a in the main text. Thus, the format is identical with that of Figure 3a. Simply, added orange squares, which is added to original plot, indicates human use of accuracy cue quantified by the same method with ideal-learner (Not HRM).

**Replication of the ideal-learner and HRM analyses with two independent parameters for the number cue**

In the main analyses, we used a single parameter for the number accuracy cue ($\beta_{n}$) by assuming that the number increase from 1 to 2 and that from 2 to 3 induce the same amount of change in acceptance degree. Assuming alternatively, i.e., differing effects between the number increase from 1 to 2 and that from 2 to 3, we modified the original model such that the single parameter for the number accuracy cue ($\beta_{n}$) was replaced with two parameters, one for the number accuracy of ‘1’ (n=1) and the other for that of ‘3’ (n=3):

$W_{ij}=W_{0}+\beta_{cm} {c^{m}}_{ij}+\beta_{n1} {{n1}^{o}}_{ij}+ \beta_{n3} {{n3}^{o}}_{ij}+ \beta_{a} {a^{o}}_{ij} +\beta_{co} {c^{o}}_{ij}$*,*

where ${n1}^{o}$ and ${n3}^{o}$ are indicator variables that indicate whether the number of others’ SP estimates is ‘1’ and ‘3’, respectively, and therefore $\beta_{n1}$ and $\beta_{n3}$ correspond to the changes of acceptance degree as the number of others’ SP estimates decreases from 2 to 1 and increases from 2 to 3, respectively. The results from this alternative set of parameters are summarized in the last rows of **Table S2A** (the ideal-learner analysis) and **Table S2B** (the HRM analysis). The result from the ideal-learner analysis was consistent with a previous study (Mannes, 2009): the ideal amount of change in acceptance degree was greater when the number changes from 2 to 1 (${\beta_{n1}}^{*}$ = -0.171) than when from 2 to 3 (${\beta_{n3}}^{*}$ = 0.079). When these values were combined with the parameter value for overall weight ($W_{0}$ = 0.665), the resultant degrees of acceptance would be 0.494 (n=1), 0.665 (n=2) and 0.744 (n=3), which are close to the known values of normative (e.g., averaging) weighting, 0.5, 0.667 and 0.75. Likewise, the HRM analysis indicated that human participants also changed the degree of acceptance more greatly when the number of others’ SP estimates decreased from 2 to 1 ($\beta_{n1}=-0.102$) than when it increased from 2 to 3 ($\beta_{n3}=0.056$).

**Table S2A. Parameters of hypothetical learner (noise-free, no individual difference)**

|  | Overall weight | Modulatory effect | | | | | Constant shift |
| --- | --- | --- | --- | --- | --- | --- | --- |
|  |  | My confidence | Number | | Agreement | Other’s confidence |  |
| Parameter | ${W_{0}}^{*}$ | ${\beta_{cm}}^{*}$ | ${\beta_{n}}^{*}$ | | ${\beta_{a}}^{*}$ | ${\beta_{co}}^{*}$(other) | $B^{*}$ |
| Ideal learner (original) | 0.637 | -0.049 | 0.257 | | 0.071 | 0.026 | 0.949 |
| Noise free human learner | 0.491  [0.470,  0.514] | -0.109 [-0.145, -0.062] | 0.173  [0.128,  0.225] | | 0.241  [0.191,  0.295] | 0.001  [-0.040,  0.042] | 0.335  [0.275,  0.398] |
| Ideal learner  (number-extended) | 0.665 | -0.039 | ${\beta_{n1}}^{*}$ | ${\beta_{n3}}^{*}$ | 0.080 | 0.032 | 0.961 |
|  |  |  | -0.171 | 0.079 |  |  |  |

**Table S2B. Parameters of behavior model (HRM)**

|  | Overall weight | Modulatory effect | | | | | Constant shift |
| --- | --- | --- | --- | --- | --- | --- | --- |
|  |  | My  confidence | Number | | Agreement | Other’s confidence |  |
| Parameter | $W_{0}$ | $\beta_{cm}$ | $\beta_{n}$ | | $\beta_{a}$ | $\beta_{co}$ | $B$ |
| Original model | 0.491 [0.450,  0.532] | -0.103  [-0.150,  -0.056] | 0.168  [0.114,  0.222] | | 0.150  [0.095,  0.205] | -0.012  [-0.060,  0.036] | 0.354  [0.249,  0.461] |
| Number variable extended | 0.505,  [0.457,  0.553] | -0.104  [-0.153,  -0.056] | $\beta_{n1}$ | $\beta_{n3}$ | 0.152  [0.098,  0.207] | -0.013  [-0.061,  0.036] | 0.352  [0.247,  0.458] |
|  |  |  | -0.102  [-0.150,  -0.055] | 0.056  [0.001,  0.112] |  |  |  |
