# Supplementary material for: Utility and use of accuracy cues in social learning of crowd preferences
Source: PLoS One. 2020 Oct 28;15(10):e0240997. doi: 10.1371/journal.pone.0240997 (PMC7592789; doi:10.1371/journal.pone.0240997)
Supplement: S3 Text — (DOCX) [file pone.0240997.s003.docx]

S3 Text

**Utility analysis based on two independent survey results.**

We collected two separate sets of survey data, one online and the other offline, to define the ‘population preference ranking (actual SP)’ of the individual items used in the main experiment. As described in Materials and Methods, the preference ranking scores was highly correlated between the two data sets. (Spearman r = 0.885)

**Fig S3A. Consistency between preference rankings determined by two different survey data sets.**

We decided to use the online survey data set (n = 100) as the ‘actual SP’ values for the following reasons. Firstly, the individuals who participated in the online survey better represent the general population than those in the offline survey. Secondly, the scoring method used in the online survey was more similar to that used in the actual experiment than that in the offline survey. Lastly, the amount of errors of the SP predictions made by the participants in the main experiment were smaller (i.e., more accurate) when the actual SP values were defined by the online-survey data (mean absolute error = 5.23) than by the offline-survey data (mean absolute error = 5.43) (p < 0.01 Wilcox signed rank test).

Although we presented our main results using the ‘actual SP’ values that were defined by the online-survey data, we made sure that the almost same results were obtained when the “actual SP values” were defined by the offline-survey data (**Fig S3B, S3C**).

**Fig S3B. Results of error related analysis based on the offline-survey data set.** Each panel corresponds to Fig 2 panel b ~ g in the main text. The original preference ranking, determined by the online-survey data set, was replaced with the new preference ranking (actual SP values) determined by the offline-survey data set. (*** p < 0.001, ** p < 0.01, * p < 0.05, · p < 0.1)

As shown in Fig S3B, we could replicate our findings about the relationship between accuracy (error) and cues using the SP values defined by the offline-survey data set. Although the overall amount of absolute errors was higher for the offline-survey data set than the original (online-survey) data set, the relative relationship and significance levels are similar between the two data sets. So, our main claim, which was that "every cue has information about accuracy, but only the number cue has significant relationship with relative accuracy", was held true in the offline-survey data set.


**Fig S3B. Results of ideal-learner analysis based on the offline-survey data set.** This is a modified version of Fig 3a in the main text. Orange circles are the parameters of the HRM and green circles are the original parameters of the ideal-learner, which are redrawn here for comparison from the original plot in the main text (Fig 3a). The additional green triangles are the parameters of the ideal-learner that were estimated by using the actual SP values defined by the offline-survey data set. Note that the green circles and green triangles are highly consistent with one another.

We also fitted the ideal learner's parameters using the actual SP values that were defined by the offline-survey data set, and the results are summarized in Fig S3B. The fitted parameters were highly consistent with those of the main analysis. A slight difference from the main analysis was found for the utility of confidence ($\beta_{cm},\beta_{co}$), whereby the parameters becomes closer to 0. In sum, the main results of the ideal-learner analysis were held true regardless of whether the actual SP values were defined by the online-survey data set or by the offline-survey data set.
