# Supplementary material for: Utility and use of accuracy cues in social learning of crowd preferences
Source: PLoS One. 2020 Oct 28;15(10):e0240997. doi: 10.1371/journal.pone.0240997 (PMC7592789; doi:10.1371/journal.pone.0240997)
Supplement: S4 Text — (DOCX) [file pone.0240997.s004.docx]

S4 Text

**Weight on advice analysis**

Some previous studies on advice taking quantified the extent to which people modulate the degree of acceptance with an index called “weight on advice (WOA)” (Bonaccio & Dalal, 2006):

$WOA=\frac{Judge final estimate-Judge initial estimate}{Advisor recommendation- Judge initial estimate}$,

which normalizes the amount of estimation change with the difference between an initial estimate and recommended estimate. In principle, this WOA method can be applied to our data as follows:

${WOA}_{ij}=\frac{{\hat{SP}^{r}}_{ij}- {\hat{SP}^{m}}_{ij}}{{\hat{SP}^{o}}_{ij}- {\hat{SP}^{m}}_{ij}}$,

where ${WOA}_{ij}$ is an WOA index computed for an item $j$ and a participant $i$. We did not choose the WOA analysis as a main analysis for two reasons. First, when the WOA indices were computed for our data, the denominator,${\hat{SP}^{o}}_{ij}- {\hat{SP}^{m}}_{ij}$, was too small (${\hat{SP}^{o}}_{ij}- {\hat{SP}^{m}}_{ij}\leq1$) in a large fraction of trials (18%), and the resultant indices also turned out of the appropriate range ($0\leq WOA\leq1$) in even a larger fraction of trials (34%). These two fractions were way greater than those reported previously (e.g., below 10% for both fractions in (Gino & Moore, 2007)). Second, it was difficult to build (hierarchical) regression model because appropriate shape of noise term is hard to be defined for the dependent variable (WOA): ratio of difference between bounded values. (we assumed noise of $\hat{SP}^{r}$ is beta-binomial in main model). Despite these limitations, we examined whether our main findings regarding human use of the accuracy cues can be replicated. In doing so, we modified the WOA analysis to address the aforementioned limitations as follows: first, the trials in which the absolute value of the denominator was smaller than 1 were discarded as WOA indices were unreliable; second, the resultant WOA indices were rectified to stay within the appropriate range ($0\leq WOA\leq1$) by converting the WOA indices smaller than 0 and greater than 1 to the value of 0 and 1, respectively, in following a previous study (Gino & Moore, 2007).

We applied this modified WOA analysis either to the entire trials or to the trials in which only a single SP estimate of others (n=1). The results are summarized in Table S3. For each of the accuracy cue, we computed the mean WOA for the different discretized levels of the cue that were used in the regression analyses.

**Table S4A. Results of the WOA analysis**

| Accuracy cue | Own confidence | | Other’s confidence | | Agreement among others | | Number of others | | |
| --- | --- | --- | --- | --- | --- | --- | --- | --- | --- |
| Value of cue | Low | High | Low | High | Low | High | 1 | 2 | 3 |
| Mean WOA  (Whole trial) | 0.523 | 0.452 | 0.499 | 0.486 | 0.459 | 0.564 | 0.453 | 0.502 | 0.520 |
| Mean WOA (1:1 integration) | 0.477 | 0.438 | 0.453 | 0.449 | - | - | - | - | - |

Mean WOA significantly differed between the different levels for own confidence, agreement among others, number of others (two-sample t-test, p < 0.05), the difference between the mean WOA was not significantly different between groups by others’ confidence. When the analysis was confined to the trials in which only a single SP estimate of others (n=1; the bottom row of Table S4A), the mean WOA significantly differed between the levels for the own (my) confidence cue (two sample t test, t = 2.14, p<0.05) but not for the others’ confidence cue (t = 0.25, p>0.1).

To further check the consistency between the results from the WOA analysis and those form the HRM analysis, we carried out a regression analysis by regressing the WOA indices onto the accuracy cues in a way similar to that for the HRM analysis:

- ${WOA}_{ij}={WOA}_{0}+ r_{cm} {c^{m}}_{ij}+r_{n} {n^{o}}_{ij} + r_{ao} {a^{o}}_{ij} + r_{co} {c^{o}}_{ij} +\epsilon_{ij}$.

In overall, the estimated parameters (${{WOA}_{o}=.496, r}_{cm}=-0.071$,$r_{n}=0.072$, $r_{ao}=0.106$, $r_{co}=-0.017$) were consistent with the corresponding parameters estimated in the HRM analysis ($W_{0}{,\beta}_{cm}$,$\beta_{n}$, $\beta_{ao}$, $\beta_{co}$): all the parameters significantly deviated from zero (p<0.001) except for that for the others’ confidence cue (p>0.1). Put together, we conclude that the WOA analysis replicated our main findings regarding human use of the accuracy cue based on the HRM analysis.

**Reference**

Bonaccio, S., & Dalal, R. S. (2006). Advice taking and decision-making: An integrative literature review, and implications for the organizational sciences. *Organizational Behavior and Human Decision Processes*, *101*(2), 127–151. https://doi.org/10.1016/j.obhdp.2006.07.001

Gino, F., & Moore, D. A. (2007). Effects of task difficulty on use of advice. *Journal of Behavioral Decision Making*, *20*(1), 21–35. https://doi.org/10.1002/bdm.539
