# Supplementary material for: Utility and use of accuracy cues in social learning of crowd preferences
Source: PLoS One. 2020 Oct 28;15(10):e0240997. doi: 10.1371/journal.pone.0240997 (PMC7592789; doi:10.1371/journal.pone.0240997)
Supplement: S1 Table — (DOCX) [file pone.0240997.s005.docx]

S1 Table

**Conditions of exposing others’ SP estimates**

| Participant’s ID  # of others’  SP estimates | **A** | **B** | **C** | **D** |
| --- | --- | --- | --- | --- |
| **1** | B | C | D | A |
|  | C | D | A | B |
|  | D | A | B | C |
| **2** | B, C | C, D | D, A | A, B |
|  | C, D | D, A | A, B | B, C |
|  | B, D | C, A | D, B | A, C |
| **3** | B, C, D | A, C, D | A, B, D | B, C, D |
| **0** | . | . | . | . |

Each row of the table indicates corresponds to one condition (type of trial), for example 4^th^ row (yellow marked) indicates participant A is exposed to participant B and participant C’s estimates, at the same time (trial), subject B is exposed to subject C and D’s estimates and etc.

In each given genre (24 trials), Trials correspond to each 1^st^ ~ 6^th^ row were appeared twice.

Trials correspond to each 7^th^ and 8^th^ row, which participants exposed to 3 other people or not exposed any of others, was appeared 6 times.
