# Supplementary material for: Utility and use of accuracy cues in social learning of crowd preferences
Source: PLoS One. 2020 Oct 28;15(10):e0240997. doi: 10.1371/journal.pone.0240997 (PMC7592789; doi:10.1371/journal.pone.0240997)
Supplement: Attachment — Submitted filename: Response_to_reviewer_fin0.docx [file pone.0240997.s006.docx]

Dear Editors:

First of all, we thank the reviewers for their constructive comments on the previously submitted version of our manuscript. Our study on those comments led us (1) to collect further data, (2) to validate the assumptions of our current statistical analyses, (3) to modify our analyses if necessary, (4) to acquire a new set of results with the newly added data, and (5) to draw new interpretations and conclusions. Accordingly we revised our manuscript based on (1)~(5). In below, we indicated what the original comments were, which were labeled as C#-#, and detailed how we addressed each of those comments in our revised manuscript, which were labeled as R#-#. Thanks to the reviewers’ critical comments, our manuscript has reached a more meaningful set of conclusions on more solid empirical and methodological grounds.

**C1**: Reviewer #1’s overall comments on the general contributions of our work

*“This paper studies how the opinion of others affect of our rankings. The authors consider an experiment in which people are asked to judge paintwork according to their "social ranking" and then exposed to rankings/opinions by others, including confidence of others. The paper shows that people adapt their opinions when receiving information. The manuscript is well-written and the experiment is well-executed in my view.”*

**C2**: Reviewer #1’s overall comments on the general contributions of our work

*“The paper asks both how people should and actually do incorporate other people's beliefs into their beliefs of others. A contribution of the paper is to extend previous work on beliefs that are "factually correct" to beliefs that are "socially correct".”*

**R1**: Our reply to **C1 and C2**

We thank the reviewers for appreciating our work’s contributions to the field. As described in what follows, we’ve now reached a conclusion that is different from the previous one, especially about the utility of ‘confidence’, based on new independent measures of ‘actual shared preference (SP) values.’ We believe that, with this revision, our manuscript makes more solid contributions to the understanding of how other people’s opinions affect our initial judgements on socially correct problems.

**C2-1**: Reviewer #2’s comment on the appropriateness of the actual SP values used in the previously submitted manuscript

*“While I like the research question and theoretical analysis, I see a major flaw in the experimental design that renders most empirical conclusions uncertain. The empirical analysis hinges on how well the experimental subjects -- students -- predict how actual people ranked paintings. However, the experiment deceived students: actual people never ranked the paintings. So in the empirical analysis the paper replaces this actual ranking with students' predictions of it: unfortunately, we do not know whether these predictions are any good. Because the empirical analysis would need these actual rankings, but has only the predicted ones, the empirical conclusions are uncertain. Unless the authors can produce such an actual ranking and then use this data to redo the analysis, I cannot recommend publication.”*

**R2-1**: Our reply to **C2-1**

We agree with the reviewer #2 on this matter. Although we initially assumed that “students’ predictions of SP” and (b) “actual SP values” are similar based on previous studies (Leder et al., 2016; Lusk & Norwood., 2009a; 2009b)—as we wrote in Materials and Methods in our previously submitted manuscript (line 734~748), the reviewer’s comment made us cast doubt on this assumption. Especially given the possibility that the validity of the assumption might depend on the domains of problems (e.g., Finnas (1987) reported a substantial discrepancy between people’s predictions of SP and actual SP on the matters of music preference), we felt we should empirically acquire the actual SP values from an independent population of participants. Thus, we collected personal preferences—not predictions of SP values—about the artworks used in our experiment from two independent sets of populations and defined the actual SP values based on these new data sets. One data set was acquired through an ‘online survey’ from 100 individuals, and the other through an ‘offline (face-to-face) survey’ from 225 individuals at a local museum site (we provided the details about these surveys in the Materials and Methods of the newly submitted manuscript, a subsection titled “Definition of actual SP”).

We first checked whether our initial assumption holds by comparing these newly acquired “actual SP values” with the original(predicted) SP values used in our old manuscript. As shown below, although they were indeed correlated (Spearman *r* was 0.843 between the old SPs and the online-survey SPs, and 0.775 between the old SPs and the offline-survey SPs), we acknowledge that there exist substantive differences between our old, predicted SP values and the directly acquired SP values, particularly considering that the correlation between the newly acquired sets of SP values (Spearman *r* was 0.884 between the online-survey SPs and the offline-survey SPs) was higher than the correlations of the old SP values with the newly acquired SPs.

Given these comparisons, we decided to use the online-survey data set (n = 100) as the ‘actual SP’ values for the following reasons. Firstly, the individuals who participated in the online survey better represent the general population than those in the offline survey. Secondly, the scoring method used in the online survey was more similar to that used in the main experiment than that in the offline survey. Lastly, the amount of errors of the SP predictions made by the participants in our main experiment was smaller (i.e., more accurate) when the ‘actual SP’ values were defined by the online-survey data set (mean absolute error = 5.23) than when defined by the offline-survey data set (mean absolute error = 5.43) (p < 0.01 Wilcox signed rank test). Although we presented our main results using the ‘actual SP’ values that were defined by the online-survey data set, we also made sure that the almost same results were obtained when the ‘actual SP’ values were defined by the offline-survey data set, which are described in a supplementary material in the newly revised manuscript (S3 Text).

The old results, which were based on the ‘actual SP’ values that were defined by the SPs predicted by the participants in the main experiment, and the new results, which were based on the ‘actual SP’ values that were defined by the online- and offline-survey data sets, were quite similar in terms of the informativeness of the accuracy cues (relationship between absolute errors and accuracy cues) but substantially differed in terms of the utility of the accuracy cues (relationship between relative errors and accuracy cues). To help appreciate the similarities and differences between the old and new results, we re-drew Figure 2d-g in the main manuscript respectively for each data set, as shown below (in the order of the old, online-survey, and offline-survey, with statistical test results: n.s., p > 0.1; ·, p < 0.1; *, p < 0.05; **, p < 0.01; ***, p<0.001).

The comparison across the three data sets indicates that the four accuracy cues all significantly possess the informativeness about accuracy (relationship with absolute error) regardless of which data sets were used for the definition of ‘actual SP’ values. However, whereas the four accuracy cues all had significant utilities in the old data set, only the number cue and the agreement cue showed strong and moderate degrees of utility in the new data sets. Particularly, we note that the both types of the confidence cue (*my* and others’) failed to show any significant or moderate degrees of utility.

Next, given its robustness and strength, we used the ‘number’ cue as a reference to which the ideal and human uses of the other accuracy cues (i.e., the ‘confidence’ and ‘agreement’ cues) are compared in terms of magnitude. To do so, we had to modify the modulator dummy variables assigned to the number cue in our original regression model, such that its dummy variables now match those assigned to the remaining cues (from {-1 0 1} to {-0.5, 0, 0.5} for {n=1, n=2, n=3}). With this modification of the dummy variables, we carried out the ideal learner analysis for the newly defined values of ‘actual SP’ values. The outcomes of the ideal learner analysis were highly consistent with those of the utility analyses: (1) the ideal learner’s degree of acceptance was modulated substantively by the number cue, and moderately by the agreement cue, but (2), unlike in the old set of ‘actual SP’ values, the ideal learner’s degree of acceptance was hardly modulated by the confidence cues, neither by my own confidence nor by others’ confidence. Again, to help appreciate the differences of the results across the three different definitions of ‘actual SP’ values, we juxtaposed the outcomes in the format of Figure 3a used in the main text as shown below.

In sum, by defining the ‘actual SP’ values based on the direct surveys from the independent, large group of participants as suggested by the reviewer #2, we reached a conclusion that is different from the old one—but more robust and sound one than the old one: (1) the confidence cues, both *mine* and others, have almost negligible degrees of utility, but people tend to overuse the *my* confidence cue; (2) the agreement cue has only a moderate degree of utility, which is substantively small compared to that of the number cue, but people tend to overuse the agreement cue and underuse the number cue. We reflected all the changes associated with the newly defined ‘actual SP’ values on the newly revised manuscript, including Abstract, Introduction, Results, Discussion, Materials and Methods, and the Supplemental materials.

**C2-2**: Reviewer #2’s comment on the independence of ranking scores within individuals

*“If they can produce it, then the empirical analysis would also need revision. For instance, within a person, each ranking is not independent, and so this regression assumption is in doubt (at the minimum, the paper needs to cluster standard errors by student).”*

**R2-2**: Our reply to **C2-2**

We share with the reviewer the concern about the possibility that an individual tries not to repeat the same responses, considering the literal meaning of ranking. For instance, once a given individual assigned #1 to a previous item, then she or he might try not to assign #1 to any following items. If so, a complex dependency is likely to exist in response measures for the ‘participant’ x ‘genre’ effect. Thus, we tested whether such a kind of dependency actually exists in our measurements by checking whether or not the frequency of a specific value of rank decreased once it was used in previous items in a given genre. As shown below, the frequency patterns of ranks remain almost the same when the trials were conditioned on the different vales of rank assigned in previous items.

In addition, we also tested the potential presence of dependency via simulation. Specifically, we simulated the number of re-occurrences of the rank assigned on the very first trial in the following trials. This simulation allows us to evaluate which of two scenarios—‘dependence scenario’ vs ‘independence scenario’—is more plausible in the current data. As shown below, if individuals’ ranking responses are completely dependent on one another (i.e., not to repeat the same response; ‘dependence scenario’), then the number of re-occurrences should remain zero (as indicated by the ‘+’ signs in the plot shown below). By contrast, if completely independent from one another (‘independence scenario’), the frequency of re-occurrences should follow a specific pattern (as indicated the black line in the plot shown below). We found that the observed frequency patterns of re-occurrences in our data (those in the revision round) were very close to the ‘independence scenario’ rather than the ‘dependence scenario.’

Based on these outcomes, we think that our behavior model’s assumption about the independence of our ranking measurements is not violated substantially. The participants in our study appear to treat their responses as ‘scaled measures’, rather than as a literal sense of ranking (i.e., assign values of 1-24 without repetition). The outcomes make sense because, due to the large (24) number of items and task difficulty especially in the revision round (which involves updating their own opinions by considering others’ opinions), it must be challenging for individuals to remember all the responses they made in previous trials.

We note that the reviewer suggested something in the parenthesis (“*at the minimum, the paper needs to cluster standard errors by student”*). We conjecture that this suggestion is to do with the potential presence of idiosyncratic differences in bias across individuals (e.g., some participants’ estimates are positively or negatively biased with different degrees). This is exactly the reason why we opted to use the hierarchical regression model (i.e. multilevel model) in our previously submitted manuscript, which takes into account different biases and effects of the modulators across individual participants. We note that our hierarchical regression model surely incorporates the suggestion made by the reviewer (“*at the minimum, the paper needs to cluster standard errors by student”*). To point out this issue and how we addressed this issue more explicitly, we revised the part of the manuscript where we describe the hierarchical regression model as follows:

"Second, to take into account the idiosyncratic differences in coefficient and responses bias across individuals, we built a hierarchical regression model (HRM; See S1 Text for details) by extending the basic regression model such that the coefficients of individuals were assumed to be random samples from a normal distribution with a mean, called a ‘fixed effect’."

**C2-3**: Reviewer #2’s comment on the violation of normal distribution of the ranking scores

*“Further, the data are truncated at 1 and 46 so for low and high ranking paintings the errors are not normally distributed.”*

**R2-3**: Our reply to **C2-3**We agree with the reviewer on this matter, and we had already addressed this issue by assuming that error distribution is a beta binomial distribution, instead of a normal distribution. To make more explicit this point in our revised manuscript, we revised the previous statements as follows:

“… Specifically, we assumed that such noise would be distributed across trials in a beta binomial distribution rather than in a Gaussian distribution, which is typically assumed in conventional regression models. The beta binomial distribution is appropriate for the current study because the response variable (ranking scores) is bounded [1, 24]), and because the error distribution shape tended to be skewed around the boundaries (see S1 Text for details). …”

**References**

1.	Leder H, Goller J, Rigotti T, Forster M. Private and Shared Taste in Art and Face Appreciation. Frontiers in Human Neuroscience. 2016;10. doi:10.3389/fnhum.2016.00155

2.	Lusk JL, Norwood FB. An Inferred Valuation Method. Land Economics. 2009;85: 500–514. doi:10.3368/le.85.3.500

3.	Lusk JL, Norwood FB. Bridging the gap between laboratory experiments and naturally occurring markets: An inferred valuation method. Journal of Environmental Economics and Management. 2009;58: 236–250. doi:10.1016/j.jeem.2008.12.003

4.	Finnas L. Do Young People Misjudge Each Others’ Musical Taste? Psychology of Music. 1987;15: 152–166. doi:10.1177/0305735687152004
